# Safety and efficacy studies of kyphoplasty, mesh-container-plasty, and pedicle screw fixation plus vertebroplasty for thoracolumbar osteoporotic vertebral burst fractures

**DOI:** 10.1186/s13018-021-02591-3

**Published:** 2021-07-06

**Authors:** Yimin Li, Yunfan Qian, Guangjie Shen, Chengxuan Tang, Xiqiang Zhong, Shaoqi He

**Affiliations:** Department of Orthopaedic Surgery, Third affiliated Hospital of Wenzhou Medical University, 108 WanSong Road, Ruian, Wenzhou, Zhejiang, China

**Keywords:** Spinal fracture, Pedicle screw fixation, Mesh-container, Osteoporosis, Vertebroplasty

## Abstract

**Background:**

Percutaneous kyphoplasty (PKP), percutaneous mesh-container-plasty (PMCP), and pedicle screw fixation plus vertebroplasty (PSFV) were three methods for osteoporotic vertebral burst fractures (OVBF). The purpose of the current study was to evaluate the clinical safety and efficacy of PKP, PMCP, and PSFV for OVBFs.

**Methods:**

This retrospective study included 338 consecutive patients with thoracolumbar OVBFs who underwent PKP (n = 111), PMCP (n = 109), or PSFV (n = 118) and compared their epidemiological data, surgical outcomes, and clinical and radiological features. Clinical evaluations of VAS and ODI and radiological evaluations of height restoration, deformity correction, cement leakage, and canal compromise were calculated preoperatively, postoperatively, and 2 years postoperatively.

**Results:**

Cement leakage (31/111 vs. 13/109 and 16/118, *P* < 0.05) was significantly higher in group PKP than in groups PSFV and PMCP. VAS and ODI scores improved postoperatively from 7.04 ± 1.15 and 67.11 ± 13.49 to 2.27 ± 1.04 and 22.00 ± 11.20, respectively, in group PKP (*P* < 0.05); from 7.04 ± 1.29 and 67.26 ± 12.79 to 2.17 ± 0.98 and 21.01 ± 7.90, respectively, in group PMCP (*P* < 0.05); and from 7.10 ± 1.37 and 67.36 ± 13.11 to 3.19 ± 1.06 and 33.81 ± 8.81, respectively, in the PSFV group (*P* < 0.05). Moreover, postoperative VAS and ODI scores were significantly higher in group PSFV than in groups PKP and PMCP (*P* < 0.05). However, VAS scores were not significantly different in the three groups 2 years postoperatively (*P >* 0.05). Postoperative anterior (81.04 ± 10.18% and 87.51 ± 8.94% vs. 93.46 ± 6.42%, *P* < 0.05) and middle vertebral body height ratio (83.01 ± 10.16% and 87.79 ± 11.62% vs. 92.38 ± 6.00%, *P* < 0.05) were significantly higher in group PSFV than in groups PMCP and PKP. Postoperatively, Cobb angle (10.04 ± 4.26° and 8.16 ± 5.76° vs. 4.97 ± 4.60°, *P* < 0.05) and canal compromise (20.76 ± 6.32 and 19.85 ± 6.18 vs. 10.18 ± 6.99, *P* < 0.05) were significantly lower in group PSFV than in groups PMCP and PKP.

**Conclusion:**

Despite relatively worse radiological results, PMCP is a safe and minimally invasive surgical method that can obtain better short-term clinical results than PKP and PSFV for OVBFs.

## Introduction

Owing to the demographic shift towards an older society, the annual incidence of osteoporosis and its associated fractures are increasing worldwide. Osteoporotic vertebral fractures (OVFs) can affect a patient’s quality of life, including chronic back pain, functional limitations, depression, and disability, which have become major health problems [1].

Thoracolumbar osteoporotic vertebral burst fractures (OVBFs) are severe types of OVFs. To date, the management of OVBFs has not been properly documented. However, surgical treatment of these fractures seems to reduce pain and mobilize the patients more quickly, and the hospital stay is therefore shorter in this case. Many patients with OVBFs without neurologic deficits have recently undergone kyphoplasty with good clinical and radiological results [2–4]. However, complications, such as cement leakage, loss of restored height, and kyphotic alignment after balloon deflation prior to cement injection [5–7].

To avoid these complications, percutaneous mesh-container-plasty (PMCP) [8] and pedicle screw fixation plus vertebroplasty (PSFV) [9, 10] have been developed with the advantages of reduced cement leakage, height restoration, and kyphotic angle reduction. During the cement injection process, the continuous injection causes the mesh container to produce a pressure, and cement leaks outside of the mesh container and enters the bone trabeculae. Therefore, a better inhibition ability for cement leakage can be achieved. The mesh container remains within the newly created vertebral cavity so that the balloon can be removed after deflation while preventing the vertebral body from collapsing. Thus, the virtual physiological vertebral body height and shape might be restored and preserved. Pedicle screw fixation reduced the fracture by ligamentotaxis before vertebroplasty and decreased the risk of cement leakage.

Based on these previous studies, we hypothesized that there would be differences in the clinical efficacy and safety of PKP, PMCP, and PSFV for the treatment of OVBFs. To test our hypothesis, we compared the clinical and radiological results of PKP, PMCP, and PSFV for the treatment of OVBFs.

## Methods

### Study design

**Fig. 1:**
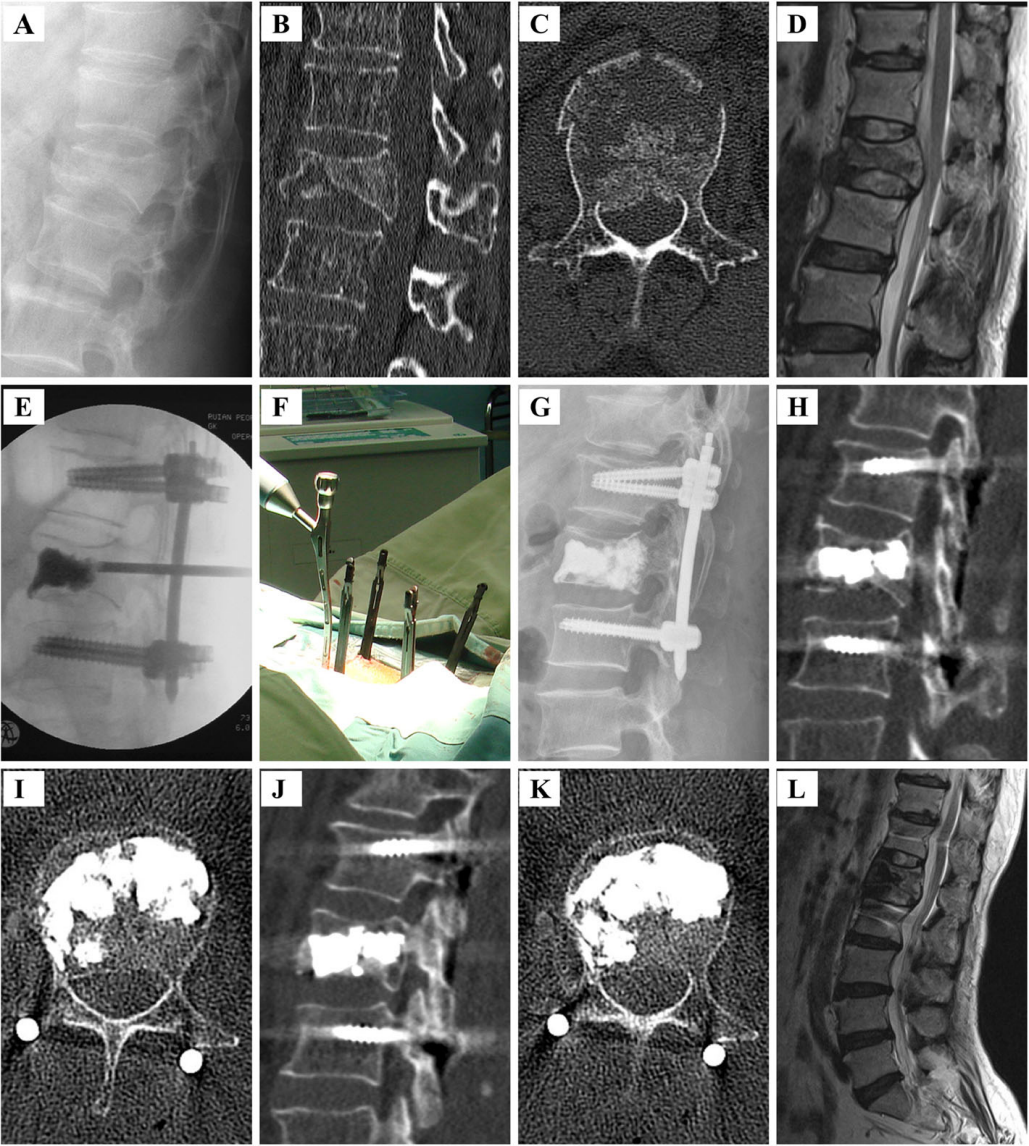
Female patient of 60 years with OVBFs in L_1_ vertebra undergoing pedicle screw fixation plus vertebroplasty. **A** Preoperative lateral radiograph showing a burst fracture of L_1_. **B**, **C** Preoperative CT-scan (plain and sagittal reconstruction image) showing the burst fracture with spinal canal compromise. **D** Preoperative MRI (T2-weighted sequences) showing the burst fracture with spinal canal compromise. **E** Intraoperative fluoroscopic image demonstrating percutaneous pedicle screws in the adjacent vertebrae and vertebroplasty in the fractured vertebra. **F** Intraoperative view. **G** Postoperative lateral roentgenogram showing adequate vertebral body reduction, excellent alignment, and reduced spinal canal encroachment following short fixation and adequate vertebral body reduction. **H**, **I** CT-scan (plain and sagittal reconstruction image) showing adequate vertebral body reduction, excellent alignment and reduced spinal canal encroachment following short fixation and adequate vertebral body reduction. **J**, **K** CT-scan (plain and sagittal reconstruction image) of the patient 1 year after surgery showing vertebral body reduction, alignment and reduced spinal canal encroachment without significant loss of correction. **L** T2-weighted sagittal MRI of the patient 4 years after surgery showing reduced spinal canal encroachment and excellent alignment and adequate vertebral body reduction

Ethical approval for this retrospective study was obtained from the Ethics Committee of the authors’ institute. We routinely obtain written informed consent for the accumulation of clinical data for future retrospective analyses from each patient who received PKP, PMCP, or PSFV at our hospital, including all patients in this study. The differences between PKP, PMCP, and PSFV were explained to all patients before surgery, and the surgical methods were selected according to patient preference. The medical records of consecutive patients who sustained OVBFs without neurologic deficit and who underwent PKP, PMCP, or PSFV from May 2015 to April 2018 were reviewed (Fig. 1).

First, we selected 434 patients who received PKP, PMCP, or PSFV. The inclusion criteria were as follows: (1) elderly (≥ 60 years), (2) thoracolumbar (T10 to L2) single fresh complete burst fracture (type A3 or A4 according to AOSpine thoracolumbar spine injury classification system) [11], (3) without neurological deficit, and (4) diagnosed with osteoporosis according to a T value of less than − 2.5 in the dual-energy X-ray absorptiometry (DXA). We then excluded patients with polytrauma and other OVFs, and those with symptoms of neurological deficits, preexisting spinal deformity or previous spinal operation, metastatic bone tumor or multiple myeloma, systemic or local infections, and severe bleeding disorders. Finally, we analyzed 338 patients who were divided into three groups according to surgical techniques: PKP (n = 111), PMCP (n = 109), and PSFV (n = 118) groups.

Preoperatively, standard clinical examination and evaluation, including medical history, physical examination of percussion pain, assessment of pain intensity (visual pain analog scale [VAS]), and activity level (Oswestry Disability Index [ODI]) [12] were evaluated. Radiographs of the relevant spinal region in two planes, computed tomography (CT) scan, magnetic resonance imaging (MRI) (T1-weighted and T2-weighted sequences including short tau inversion recovery sequences), and DXA were performed. All patients received calcium supplementation (1000 mg of elemental calcium daily) and vitamin D (400–600 IU). Twenty-six patients in the PKP group, 20 in the PMCP group, and 27 in the PSFV group received hormonal replacement therapy (estrogen and progestin). Bisphosphonates were administered to 97 patients (zoledronate, n = 29; alendronate, n = 68) in the PKP group, 89 patients (zoledronate, n = 25; alendronate, n = 64) in the PMCP group, and 91 patients in the PSFV group (zoledronate, n = 33; alendronate, n = 58).

### Surgical technique

Three independent spine surgeons performed surgeries for PKP, PMCP, or PSFV. In the PSFV group, all surgical procedures were performed under general anesthesia with endotracheal intubation. Patients were placed in a prone position on four bolsters placed on a radiolucent operating table with the abdomen freely suspended. Patients were positioned with surgical bolsters placed under the thorax and iliac crests to induce spinal lordosis and facilitate fracture reduction. Percutaneous pedicle screw fixation was performed using a Zina™ device (Sanyou Medical Co., Ltd, Shanghai, China) under anteroposterior and lateral fluoroscopic views. A targeting cannulated needle (Sanyou Medical Co., Ltd, Shanghai, China) for each pedicle of the instrumented adjacent vertebrae was used to locate the pedicle. K-wires (2 mm) were then passed through the needle. After removal of the targeting needle, cannulated pedicle screws were placed with extender sleeves down into the pedicles of the non-fractured vertebrae above and below the fractured vertebra, and all 4 K-wires were subsequently removed. A 6-mm-diameter trocar (Dragon Crown Medical Co., Ltd., Jinan City, Shandong Province, China) was inserted, followed by a cannula into the intact pedicle at the fractured vertebra. The position was controlled by an image intensifier, which was then enlarged using an access cannula with a trocar. Once the cannula reached the optimal position, the trocar was removed, and polymethylmethacrylate (PMMA) cement was injected into the defect of the fractured body through the cannula under continuous fluoroscopic monitoring (Fig. 1). PMMA insertion was considered complete when it reached the posterior third of the vertebral body.

**Fig. 2:**
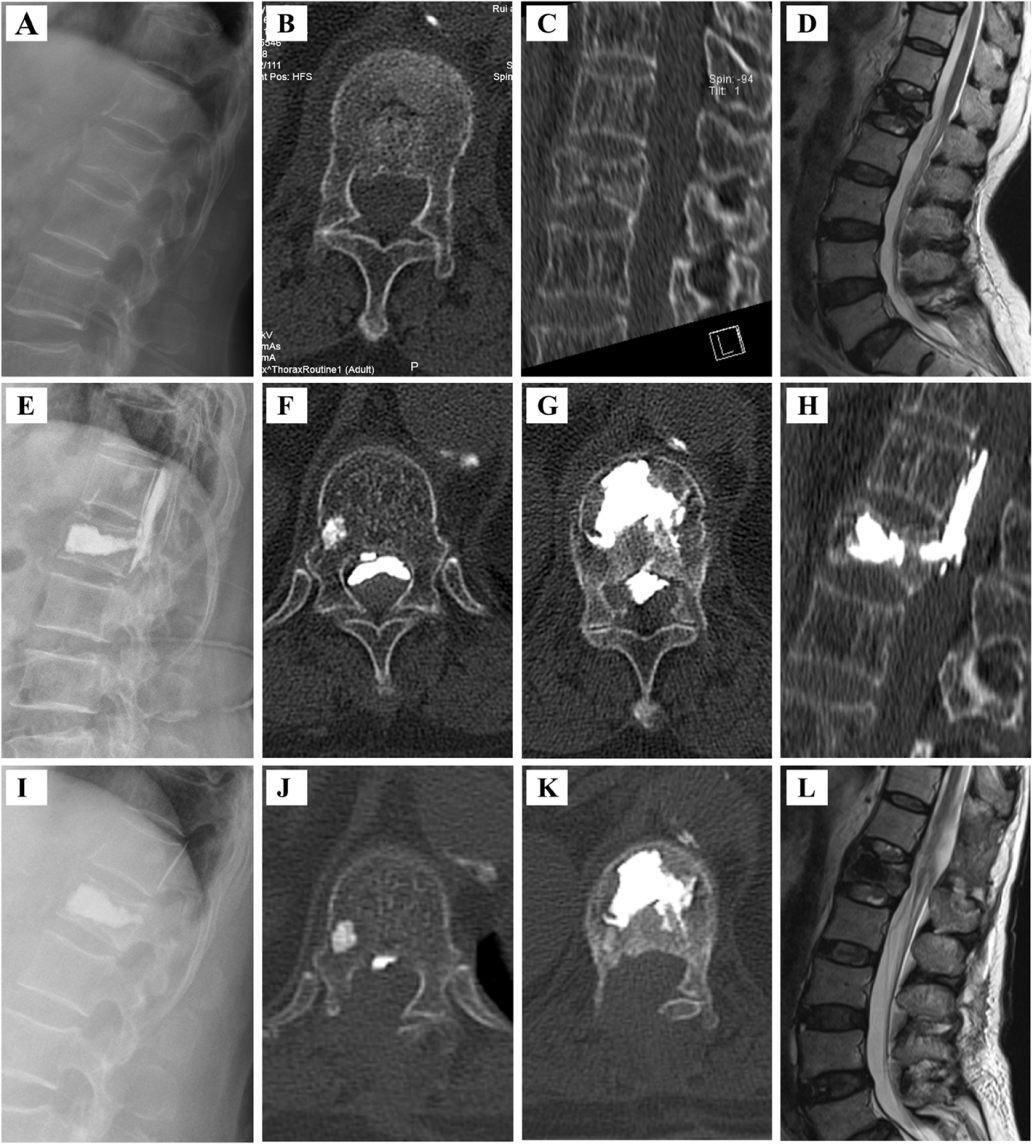
PKP surgical procedure for the treatment of a 69-year-old female patient with OVBFs in L_1_ vertebra. **A** Preoperative lateral radiograph showing a burst fracture of L_1_. **B**, **C** Preoperative CT-scan (plain and sagittal reconstruction image) showing the burst fracture with spinal canal compromise. **D** Preoperative MRI (T2-weighted sequences) showing the burst fracture with spinal canal compromise. **E** Postoperative lateral radiograph showing cement leaking from T_12_ to L_1_. **F**, **G**, **H** Postoperative CT-scan (plain at T_12_ and L_1,_ and sagittal reconstruction image) showing cement leaking with spinal canal compromise. **I** Lateral radiograph showing removed cement leaking. **J**, **K** CT-scan (plain at T_12_ and L_1_) showing removed cement leaking. **L** MRI (T2-weighted sequences) 2 years after surgery showing reduced spinal canal compromise

In the PKP and PMCP groups, all surgical procedures were performed under local anesthesia. Patients were positioned in a prone position on four bolsters placed on a radiolucent operating table with the abdomen freely suspended. A 1-cm skin incision was made lateral to the desired entry point of the pedicle percutaneously. A trocar (Dragon Crown Medical Co., Ltd., Jinan City, Shandong Province, China) in a cannula was inserted into the pedicle at the fractured vertebra through the pedicular approach as a working channel. After removing the trocar, a balloon was placed into the working channel and slowly inflated to create a low-pressure cavity for cement injection. The inflation continued until the balloon pressure reached 300 psi. If the anteroposterior radiograph revealed that the balloon exceeded the midline of the vertebra, the balloon was deflated and removed. If the balloon did not exceed the midline of the vertebra, a bilateral puncture was required, and the balloon was deflated and removed. PMMA cement was then manually injected into the vertebral body under fluoroscopic guidance (Fig. 2).

**Fig. 3:**
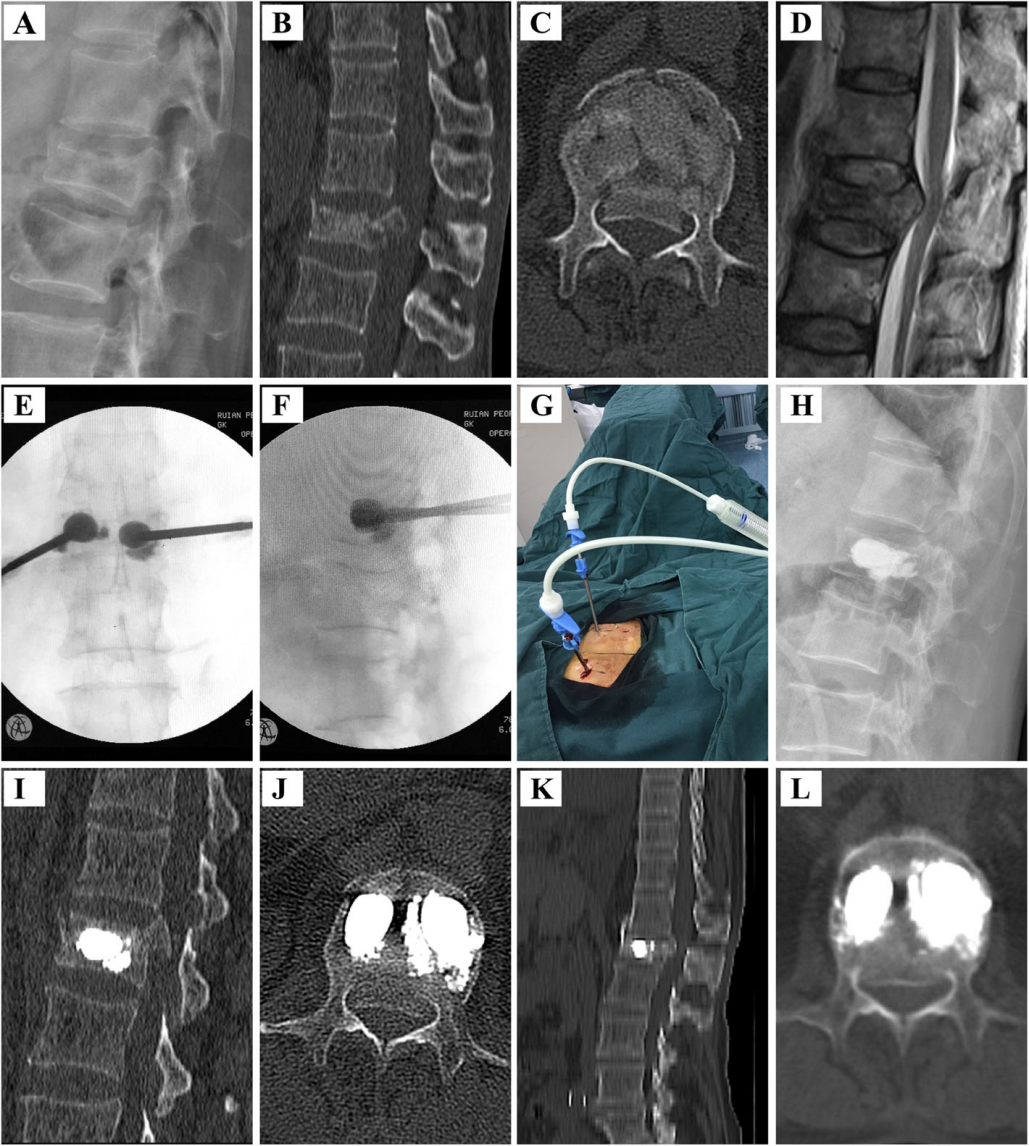
PMCP surgical procedure for the treatment of a 65-year-old female patient with OVBFs in L_1_ vertebra. **A** Preoperative lateral radiograph showing a burst fracture of L_1_. **B**, **C** Preoperative CT-scan (plain and sagittal reconstruction image) showing the burst fracture with spinal canal compromise. **D** Preoperative MRI (T2-weighted sequences) showing the burst fracture with spinal canal compromise. **E**, **F** Intraoperative fluoroscopic image demonstrating PMCP surgical procedure. **G** Intraoperative view. **H** Postoperative lateral radiograph showing better alignment following cement injection and adequate vertebral body reduction. **I**, **J** Postoperative CT-scan (plain and sagittal reconstruction image) showing no worse spinal canal compromise and better alignment and adequate vertebral body reduction. **K**, **L** CT-scan (plain and sagittal reconstruction image) 2 years after surgery showing reduced spinal canal compromise, excellent fracture healing and alignment

In the PMCP group, a mesh container made of polyethylene terephthalate (PET) (Dragon Crown Medical Co., Ltd., Jinan City, Shandong Province, China) was advanced into the cavity. Then, PMMA cement was manually injected into the mesh container within the treated vertebral body by applying a cement perfusion apparatus under fluoroscopic guidance. With the continuous injection of PMMA, the mesh container was inflated, and the height of the fractured vertebra was restored. At a certain injection amount, PMMA cement leaked outside the mesh container from the meshes and entered the bone trabeculae, which was considered complete when it reached the posterior third of the vertebral body or had a potential tendency of cortical, epidural, and anterior venous cement leakage (Fig. 3).

The operation time, estimated blood loss, and PMMA volume were recorded. All patients were followed clinically and radiographically at 1 day, 3 months, and 6 months after surgery and at every 1-year interval thereafter. Patients were assessed for neurological complications. All patients underwent CT after surgery and 1 and 2 years later. Anterior and middle vertebral body height ratios (AVBHr and MVBHr) and segmental kyphosis were measured using lateral radiographs. Canal compromise was measured using CT images. Cement leakage was determined using CT images of all sections of the fractured vertebra. Back pain intensity was recorded using VAS. Functional outcomes were measured using ODI. Three independent blinded spine surgeons completed the clinical evaluation of the patients. Additionally, two other independent blinded spine surgeons assessed the radiographs.

### Statistical analysis

SPSS 18 was used for statistical analysis. Continuous variables are expressed as mean ± standard deviation. Statistical analysis was performed for changes in each radiographic and functional parameter. Independent data, including age, body mass index (BMI), T-score, injury time, operation time, blood loss, hospital day, cost, and injected cement volume, were compared between the PMCP and PSFV groups using one-way analysis of variance (ANOVA). Differences in sex, distribution of fractured vertebrae, and cement leakages between the two groups were compared using the chi-square test. Three-way repeated measures ANOVA was used to compare VAS, ODI, AVBHr, MVBHr, canal compromise, and Cobb angle between the three groups. Statistically significant differences were defined at a 95% confidence level.

## Results

The clinical characteristics of the 338 patients are summarized in Table 1. There were no statistical differences in demographic data, including age, sex, distribution of fractured vertebrae, T-score, BMI, and injury time between these three groups (*P >* 0.05). The cost in group PMCP (4.82 ± 0.21) was significantly lower than that in group PSFV (5.50 ± 0.29, *P* < 0.05) and higher than that in group PKP (3.30 ± 0.25, *P* < 0.05). The mean operation time (92.70 ± 17.24 vs. 34.35 ± 8.72 and 31.83 ± 4.12, *P* < 0.05), blood loss (22.52 ± 4.79 vs. 7.36 ± 3.67 and 7.20 ± 2.06, *P* < 0.05), hospital stay (5.46 ± 2.31 vs. 4.42 ± 1.62 and 4.24 ± 1.62, *P* < 0.05) were significantly higher in group PSFV than in groups PMCP and PKP. There were no statistically significant differences in cement volume between the three groups. The details are presented in Table 2.

**Table 1:** Basic characteristics and comparative analysis between group PKP, PMCP, and PSFV for the treatment of the 338 patients with thoracolumbar OVBF in this study

	PKP(n = 111)	PMCP (n = 109)	PSFV(n = 118)	*F/χ*^2^	*P*
Age (years)	71.48 ± 7.01	73.29 ± 8.07	72.72 ± 8.03	1.597	0.204
Male/female	25/86	34/75	39/79	3.457	0.178
Distribution				4.235	0.835
T10	10	14	18		
T11	12	12	17		
T12	31	30	31		
L1	37	31	29		
L2	21	22	23		
Fracture type					
A3	57	63	67	2.257	0.324
A4	54	46	51		
T-score	− 3.06 ± 0.39	−3.10 ± 0.44	−3.17 ± 0.48	1.738	0.178
BMI	24.11 ± 4.44	23.22 ± 4.20	23.14 ± 4.09	1.799	0.167
Injury time (days)	4.15 ± 2.32	4.18 ± 2.09	4.05 ± 1.85	0.127	0.881

*PMCP* percutaneous mesh-container-plasty, *PSFV* pedicle screw fixation plus vertebroplasty, *OVBF* osteoporotic vertebral burst fractures, *BMI* body mass index

**Table 2:** Patient’s perioperative parameters comparison between groups PKP, PMCP, and PSFV for the treatment of the 338 patients with thoracolumbar OVBF in this study

	PKP (n = 111)	PMCP (n = 109)	PSFV (n = 118)	*F/χ*^2^	*P*
Operation time(min)	31.83 ± 4.12	34.35 ± 8.72	92.70 ± 17.24	999.476	<0.001
Blood loss(ml)	7.20 ± 2.06	7.36 ± 3.67	22.52 ± 4.79	649.273	<0.001
Hospital day(days)	4.24 ± 1.62	4.42 ± 1.62	5.46 ± 2.31	13.876	<0.001
Cost(thousand dollar)	3.30 ± 0.25	4.82 ± 0.21	5.50 ± 0.29	2227.527	< 0.001
Cement leakages	31/111	13/109	16/118	11.827	0.003
Cement volume(ml)	6.61 ± 1.72	6.19 ± 1.74	6.22 ± 1.96	999.476	0.160

*PMCP* percutaneous mesh-container-plasty, *PSFV* pedicle screw fixation plus vertebroplasty, *OVBF* osteoporotic vertebral burst fractures

### Clinical evaluation

VAS scores were reduced from preoperative 7.04 ± 1.15 to postoperative 2.27 ± 1.04 (*P <* 0.05) and 2 years postoperative 1.87 ± 0.84 in group PKP; from preoperative 7.04 ± 1.29 to postoperative 2.17 ± 0.98 (*P <* 0.05) and 2 years postoperative 1.76 ± 0.83 in group PMCP; and from preoperative 7.10 ± 1.37 to postoperative 3.19 ± 1.06 (*P <* 0.05) and 2 years postoperative 1.71 ± 0.95 in group PSFV. The ODI scores improved from preoperative 67.11 ± 13.49 to postoperative 22.00 ± 11.20 and 2 years postoperative 16.18 ± 9.11 in the PKP group (*P <* 0.05); from preoperative 67.26 ± 12.79 to postoperative 21.01 ± 7.90 and 2 years postoperative (16.40 ± 7.29) in the PMCP group (*P <* 0.05); and from preoperative 67.36 ± 13.11 to postoperative 33.81 ± 8.81 and 2 years postoperative 15.47 ± 7.65 in the PSFV group (*P* < 0.05). Moreover, VAS and ODI scores were significantly higher in the PSFV group than in both the PKP and PMCP groups postoperatively (*P* < 0.05). However, there was no difference in VAS scores between the three groups 2 years postoperatively (*P >* 0.05). The details are presented in Table 3.

**Table 3:** Clinical comparisons group PKP, PMCP, and PSFV for the treatment of the 338 patients with thoracolumbar OVBF in this study

	PKP (n = 111)	PMCP (n = 109)	PSFV (n = 117)	*F*	*P*
VAS					
Preoperative	7.04 ± 1.15	7.04 ± 1.29	7.10 ± 1.37	0.100	0.904
Postoperative	2.27 ± 1.04	2.17 ± 0.98	3.19 ± 1.06	34.578	< 0.001
2 years postoperative	1.87 ± 0.84	1.76 ± 0.83	1.71 ± 0.95	1.014	0.364
ODI					
Preoperative	67.11 ± 13.49	67.26 ± 12.79	67.36 ± 13.11	0.010	0.990
Postoperative	22.00 ± 11.20	21.01 ± 7.90	33.81 ± 8.81	65.973	< 0.001
2 years postoperative	16.18 ± 9.11	16.40 ± 7.29	15.47 ± 7.65	0.416	0.660

*PMCP* percutaneous mesh-container-plasty, *PSFV* pedicle screw fixation plus vertebroplasty, *OVBF* osteoporotic vertebral burst fractures, *VAS* visual pain analog scale, *ODI* Oswestry Disability Index

Therefore, compared with the PSFV group, the PKP and PMCP groups had better short-term pain relief and functional recovery. However, there was no difference in long-term pain relief among the three groups.

### Radiologic evaluation

AVBHr, MVBHr, and Cobb angle were improved from preoperative 65.10 ± 10.54%, 71.87 ± 11.49%, and 13.33 ± 4.26°, respectively, to postoperative 81.04 ± 10.18%, 83.01 ± 10.16%, and 10.04 ± 4.26°, respectively, in group PKP (*P <* 0.05); from preoperative 64.88 ± 11.02%, 71.00 ± 12.57%, and 13.51 ± 5.64°, respectively, to postoperative 87.51 ± 8.94%, 87.79 ± 11.62%, and 8.16 ± 5.76°, respectively, in group PMCP (*P <* 0.05); and from preoperative 64.60 ± 9.02%, 70.81 ± 7.88%, and 13.44 ± 5.07°, respectively, to postoperative 93.46 ± 6.42%, 92.38 ± 6.00, and 4.97 ± 4.60°, respectively, in group PSFV (*P <* 0.05). Moreover, long-term follow-up results showed that the AVBHr, MVBHr, PVBHr, and Cobb angle did not significantly change even 2 years postoperatively. Canal compromise was improved from preoperative (20.46 ± 7.48) to postoperative (10.18 ± 6.99) in group PSFV (*P <* 0.05). There was no difference in canal compromise between the preoperative and postoperative groups in both the PKP and PMCP groups.

Furthermore, AVBHr and MVBHr in the PMCP group were greater than those in the PKP and PMCP groups postoperatively and 2 years postoperatively (*P* < 0.05). Canal compromise and Cobb angle scores in the PSFV group were lower than those in the PKP and PMCP groups postoperatively and 2 years postoperatively (*P* < 0.05). All radiographic results are shown in Table 4.

**Table 4:** Radiologic comparisons between groups PKP, PMCP, and PSFV for the treatment of the 338 patients with thoracolumbar OVBF in this study

	PKP (n = 111)	PMCP (n = 109)	PSFV (n = 117)	*F*	*P*
AVBHr (%)					
Preoperative	65.10 ± 10.54	64.88 ± 11.02	64.60 ± 9.02	0.069	0.933
Postoperative	81.04 ± 10.18	87.51 ± 8.94	93.46 ± 6.42	60.319	< 0.001
2 years postoperative	80.77 ± 7.47	87.02 ± 8.79	93.03 ± 6.49	74.731	< 0.001
MVBHr (%)					
Preoperative	71.87 ± 11.49	71.00 ± 12.57	70.81 ± 7.88	0.311	0.733
Postoperative	83.01 ± 10.16	87.79 ± 11.62	92.38 ± 6.00	22.938	< 0.001
2 years postoperative	82.54 ± 10.30	84.49 ± 13.53	92.39 ± 6.06	189.947	< 0.001
Cobb angle (°)					
Preoperative	13.33 ± 4.26	13.51 ± 5.64	13.44 ± 5.07	0.038	0.963
Postoperative	10.04 ± 4.26	8.16 ± 5.76	4.97 ± 4.60	31.390	< 0.001
2 years postoperative	11.02 ± 4.37	8.63 ± 5.92	4.99 ± 4.66	41.991	< 0.001
Canal compromise (%)					
Preoperative	20.85 ± 6.33	20.12 ± 5.72	20.46 ± 7.48	0.342	0.711
Postoperative	20.76 ± 6.32	19.85 ± 6.18	10.18 ± 6.99	93.254	< 0.001
2 years postoperative	20.66 ± 6.24	19.90 ± 5.98	10.03 ± 7.20	95.261	< 0.001

*PMCP* percutaneous mesh-container-plasty, *PSFV* percutaneous pedicle screw fixation plus vertebroplasty, *OVBF* osteoporotic vertebral burst fractures, *AVBHr* anterior vertebral body height ratio, *MVBHr* middle vertebral body height ratio

Therefore, compared with the PKP and PMCP groups, the PSFV group had significantly higher height restoration and improvement in segmental kyphosis and canal compromise.

### Surgical complications

Cement leakage was observed in 13.69% (16/118) of patients in the PSFV group (10 in the disc or around the vertebral body through a cortical defect, 2 into the segmental vein, and 4 into the spinal canal via the basivertebral vein), 11.9% (13/109) in the PMCP group (9 into the disc or around the vertebral body through a cortical defect, 2 into the segmental vein, and 2 into the spinal canal via the basivertebral vein), and in 28.9% (31/111) in the PKP group (18 into the disc or around the vertebral body through a cortical defect, 6 into the segmental vein, and 9 into the spinal canal via the basivertebral vein, respectively(*P* > 0.05) (Table 2). A patient undergoing PKP treatment experienced bone cement leakage and nerve injury symptoms after surgery. She underwent bone cement removal and decompression surgery. However, postoperative nerve injury symptoms did not improve (Fig. 2). All other cement leakages were asymptomatic, and no surgical intervention was required to remove the extravasated cement. Postoperative complications, such as neurological functional aggravation, hemorrhage, wound healing abnormalities, infection, pulmonary embolism, and failure of posterior instrumentation were not observed during the 2-year follow-up period.

Based on the above analyses, PMCP and PSFV had significantly better safety than PKP for treating OVBFs.

## Discussion

Osteoporosis and associated fractures are prevalent in clinics, especially in women > 50 years of age. A standardized treatment strategy for osteoporotic thoracolumbar burst fractures is not currently available [2, 10, 13–15]. Percutaneous kyphoplasty (PKP) has been increasingly used in older people due to its minimally invasive nature. However, a major disadvantage of PKP is cement leakage, loss of restored height, and kyphotic alignment after balloon deflation prior to cement injection [16, 17]. Therefore, pedicle screw fixation combined with vertebroplasty and mesh containers were developed for the advantages of safety in cement leakage, height restoration, and improvement in segmental kyphosis [8, 10].

Patients with osteoporosis often present with multiple medical comorbidities and poorly endure open surgeries. Minimally invasive techniques have advantages, including preservation of back muscles, quick return to daily activities, the disappearance of pain, minimal operative risks and comorbidity, and maintenance of stability [18, 19]. In our study, the operative time, blood loss, and hospital stay were significantly lower in the PMCP group than in the PSFV group. After undergoing minimally invasive surgery, patients are able to return home quickly. For patients using PMCP as our preceding operative method, the average cost was relatively lower than that of the PSFV group.

In our study, the height restoration, improvement in segmental kyphosis, and canal compromise in the PSFV group were all higher than those in the PMCP group. Vertebral body height (AVBHr, MVBHr), segmental kyphosis, and canal compromise were significantly improved postoperatively and were stable over time, with a minimal loss of correction at 2 years postoperatively. Previous studies have indicated that PKP is ineffective for height restoration and improvement in segmental kyphosis, which was attributed to postural reduction with cement strengthening. The most significant factors affecting improvement in segmental kyphosis were fracture type and cement-injected volume [20–22].

Our previous study showed that improvement in segmental kyphosis in the PMCP group was higher than that in the PKP group with respect to both A3 and A4 fractures. The possible mechanism for height restoration and kyphosis correlation is the inflation of the mesh container. Applying pedicle-screw fixation is known to maintain restored vertebral height and involve the risk of secondary kyphosis [10, 23]. Height restoration and improvement in segmental kyphosis of the treated fractured vertebral body might be important parameters for evaluating the clinical efficacy of minimally invasive techniques. However, previous studies found no correlation between reconstitution of the vertebral body and clinical outcome (pain reduction) [24, 25]. In this study, both PMCP and PSFV treatments had significant ability in pain relief and functional recovery postoperatively and were preserved at 2 years postoperatively. However, PMCP obtained better satisfactory clinical results postoperatively than PSFV for OVBFs.

Cementoplasty involves risks of complications, including pulmonary embolism, intradiscal cement leakage, neurological deficit, paraplegia, and even death [5, 7, 26]. The risk of cement leaking into the spinal canal during classical vertebroplasty and kyphoplasty interventions is greater when the posterior wall has been damaged, as in the case of burst fractures [27]. The mesh container in the PMCP treatment keeps PMMA cement inside the container, and only partial cement leaks outside from the mesh to the bone trabeculae [8]. Reduction of the fracture by ligamentotaxis before vertebroplasty might also decrease the risk of cement leakage due to the resulting alignment of cortical bone fragments [10, 28]. After reducing the fracture using pedicle screw fixation, the reduced fracture can be consolidated by injecting cement as anteriorly as possible and stopped if the cement gets close to the posterior aspect of the vertebra or leaked into an extra osseous space, which prevents the cement from entering the spinal canal [29]. In our study, 17.43% (19/109) of the PMCP group (10 into the disc or around the vertebral body through a cortical defect, 6 into the segmental vein, and 3 into the spinal canal via the basivertebral vein) and 18.64% (22/118) of patients had PMMA leakage in the PSFV groups (13 into the disc or around the vertebral body through a cortical defect, 5 into the segmental vein, and 4 into the spinal canal via the basivertebral vein), respectively (*P* > 0.05). Therefore, PMCP treatment had similar inhibition ability of cement leakage compared with PSFV treatments.

The limitations of our study include its small patient population, short follow-up period, and retrospective design. Future studies with a prospective randomized controlled study enrolling more patients through a long-term follow-up period are needed to compare PMCP with PSFV more reliably and objectively.

## Conclusions

Despite the relatively worse radiological results, PMCP is a safe and minimally invasive surgical method that can obtain better short-term pain relief and functional recovery compared to PSFV for OVBFs.

## Data Availability

The patients’ data were collected in the Third affiliated Hospital of Wenzhou Medical University.

## Abbreviations

PMCP: Percutaneous mesh-container-plasty
PSFV: Pedicle screw fixation plus vertebroplasty
OVBFs: Osteoporotic vertebral burst fractures
PET: Polyethylene terephthalate
AVBHr: Anterior vertebral body height ratio
MVBHr: Middle vertebral body height ratio
BMI: Body mass index
PKP: Percutaneous kyphoplasty
VAS: Visual pain analog scale
ODI: Oswestry Disability Index
OVF: Osteoporotic vertebral fractures
DXA: Dual-energy X-ray absorptiometry
CT: Computed tomography
MRI: Magnetic resonance imaging
PMMA: Polymethylmethacrylate
